# Ethyl­enediammonium tetra­bromido­mercurate(II) monohydrate

**DOI:** 10.1107/S160053680902772X

**Published:** 2009-07-18

**Authors:** B. Thimme Gowda, Sabine Foro, Hiromitsu Terao, Hartmut Fuess

**Affiliations:** a Department of Chemistry, Mangalore University, Mangalagangotri 574 199, Mangalore, India; b Institute of Materials Science, Darmstadt University of Technology, Petersenstrasse 23, D-64287 Darmstadt, Germany; c Faculty of Integrated Arts and Sciences, Tokushima University, Minamijosanjima-cho, Tokushima 770-8502, Japan

## Abstract

The Hg^II^ atoms in the crystal structure of the title compound, (C_2_H_10_N_2_)[HgBr_4_]·H_2_O, are tetra­hedrally coordinated by four Br atoms and the resulting [HgBr_4_]^2−^ ions are inter­connected to the [NH_3_—CH_2_—CH_2_—NH_3_]^2+^ ions and water mol­ecules by N—H⋯Br and O—H⋯Br bonds, forming a three-dimensional network. N—H⋯O inter­actions are also present. The observed three different Hg—Br distances of 2.5597 (6), 2.6862 (8) and 2.6923 (8) Å in the tetra­bromo­mercurate unit are due to the connection of Br atoms to different numbers of H atoms. The Hg, O and two Br atoms are located on a crystallographic mirror plane. The cation has 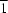 symmetry with the center of the C—C bond lying on a crystallographic center of inversion.

## Related literature

For synthetic methods, see: Furukawa *et al.* (2005 ▶). For background to Hg–halogen bonds, see: Ishihara *et al.* (2002 ▶); Furukawa *et al.* (2005 ▶). For a related structure, see: Terao *et al.* (2009 ▶).
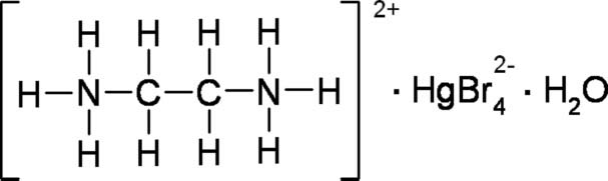


## Experimental

### 

#### Crystal data

(C_2_H_10_N_2_)[HgBr_4_]·H_2_O
*M* *_r_* = 600.37
Monoclinic, 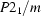

*a* = 6.4976 (6) Å
*b* = 11.416 (1) Å
*c* = 8.0161 (8) Å
β = 103.38 (1)°
*V* = 578.47 (9) Å^3^
*Z* = 2
Mo *K*α radiation
μ = 27.07 mm^−1^
*T* = 100 K
0.16 × 0.10 × 0.06 mm

#### Data collection

Oxford Diffraction Xcalibur diffractometer with a Sapphire CCD detector
Absorption correction: multi-scan (*CrysAlis RED*; Oxford Diffraction, 2009 ▶) *T*_min_ = 0.052, *T*_max_ = 0.197
2304 measured reflections
1240 independent reflections
1159 reflections with *I* > 2σ(*I*)
*R*_int_ = 0.019

#### Refinement

*R*[*F* ^2^ > 2σ(*F* ^2^)] = 0.024
*wR*(*F* ^2^) = 0.062
*S* = 1.11
1240 reflections
55 parameters
3 restraints
H atoms treated by a mixture of independent and constrained refinement
Δρ_max_ = 2.00 e Å^−3^
Δρ_min_ = −1.60 e Å^−3^

### 

Data collection: *CrysAlis CCD* (Oxford Diffraction, 2009 ▶); cell refinement: *CrysAlis RED* (Oxford Diffraction, 2009 ▶); data reduction: *CrysAlis RED*; program(s) used to solve structure: *SHELXS97* (Sheldrick, 2008 ▶); program(s) used to refine structure: *SHELXL97* (Sheldrick, 2008 ▶); molecular graphics: *PLATON* (Spek, 2009 ▶); software used to prepare material for publication: *SHELXL97*.

## Supplementary Material

Crystal structure: contains datablocks I, global. DOI: 10.1107/S160053680902772X/bt5004sup1.cif

Structure factors: contains datablocks I. DOI: 10.1107/S160053680902772X/bt5004Isup2.hkl

Additional supplementary materials:  crystallographic information; 3D view; checkCIF report

## Figures and Tables

**Table 1:** Hydrogen-bond geometry (Å, °)

*D*—H⋯*A*	*D*—H	H⋯*A*	*D*⋯*A*	*D*—H⋯*A*
N1—H1*A*⋯Br3^i^	0.91	2.56	3.359 (5)	147
N1—H1*A*⋯Br1^ii^	0.91	3.14	3.655 (5)	118
N1—H1*B*⋯O1^iii^	0.91	1.98	2.882 (5)	169
N1—H1*C*⋯Br1^i^	0.91	2.72	3.482 (4)	141
N1—H1*C*⋯Br2	0.91	2.95	3.503 (5)	121
O1—H1*O*⋯Br3^iv^	0.881 (19)	3.02 (3)	3.521 (6)	118 (2)

Symmetry codes: (i) 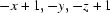; (ii) 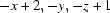; (iii) 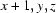; (iv) 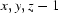.

**Fig. 1.**
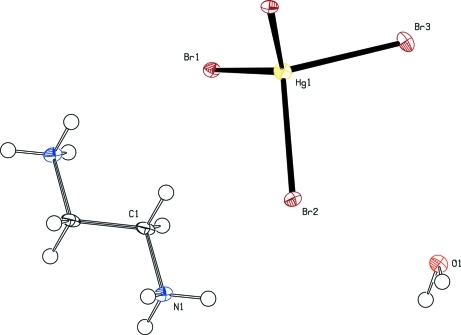
Molecular structure of (I), showing the atom labeling scheme. The displacement ellipsoids are drawn at the 50% probability level. The H atoms are represented as small spheres of arbitrary radii.

**Fig. 2.**
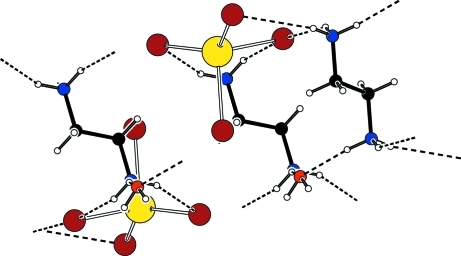
Connection scheme of the HgBr4 terahedra with the connected [NH3—CH2—CH2—NH3]2+ ions, showing the different Hg—Br bonds.

**Fig. 3.**
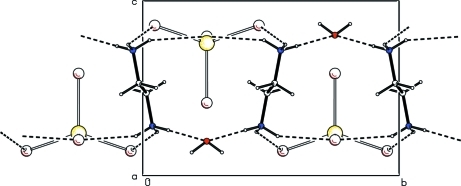
Packing diagram of (I) as viewed in the direction of a axis.

## supplementary crystallographic information

### Comment

Hg atoms due to their soft nature are amenable to polarization and thus the Hg-halogen bonds are sensitive to the intermolecular interactions such as hydrogen bonding (Ishihara *et al.*, 2002). This was evident in the halogen NQR of the Hg compounds in which the resonance frequencies are widely spread (Furukawa *et al.*, 2005). Thus the study of the structure and bonding of this class of compounds is interesting. As a part of our studies in this direction (Terao *et al.*, 2009), we report herein the crystal structure of ethylenediammonium tetrabromomercurate(II) monohydrate (I) (Fig. 1). In the structure, mercury atoms are tetrahedrally coordinated by four bromine atoms and the resulting HgBr_4_ tetrahedra are interconnected to the [NH_3_—CH_2_—CH_2_—NH_3_]^2+^ ions and water molecules by bromine-hydrogen bonds forming a three-dimensional network (Fig. 2). Three different Hg—Br distances observed [Hg—Br1 = 2.5597 (6) Å, Hg—Br2 = 2.6862 (8) Å and Hg—Br3 = 2.6923 (8) Å] establish the existence of three inequivalent Br atoms in the tetrabromomercurate unit. This may be due to the difference in intensity of N—H···Br and O—H···Br hydrogen bonding with different Br atoms. The packing diagram of the crystal structure, as viewed in the direction of *a* axis is shown in Fig. 3.

### Experimental

Ethylenediammonium tetrabromomercurate(II) monohydrate crystals were prepared by mixing equimolecular proportions of ethylenediammonium bromide and mercury(II) bomide into a methanol solution, followed by a successive evaporation of the solvent.

### Refinement

The H atom of the water molecule was located in difference map and was refined with restrained geometry, *viz*. the O—H distance was restrained to 0.85 (3) Å and H—H distance was restrained to 1.365 Å, thus leading to the angle of 107°. The other H atoms were positioned with idealized geometry using a riding model with N—H = 0.91 Å and C—H = 0.99 Å. All H atoms were refined with isotropic displacement parameters set to 1.2 times of the *U*_eq_ of the parent atom.

The residual electron-density features are located in the region of Hg1. The highest peak and the deepest hole are 0.91 and 0.71 Å from Hg1, respectivily.

### Crystal data

**Table:**

(C_2_H_10_N_2_)[HgBr_4_]·H_2_O	*F*(000) = 532
*M**_r_* = 600.37	*D*_x_ = 3.447 Mg m^−^^3^
Monoclinic, *P*2_1_/*m*	Mo *K*α radiation, λ = 0.71073 Å
Hall symbol: -P 2yb	Cell parameters from 1937 reflections
*a* = 6.4976 (6) Å	θ = 2.6–27.9°
*b* = 11.416 (1) Å	µ = 27.07 mm^−^^1^
*c* = 8.0161 (8) Å	*T* = 100 K
β = 103.38 (1)°	Prism, colourless
*V* = 578.47 (9) Å^3^	0.16 × 0.10 × 0.06 mm
*Z* = 2	

### Data collection

**Table:**

Oxford Diffraction Xcalibur diffractometer with a Sapphire CCD detector	1240 independent reflections
Radiation source: fine-focus sealed tube	1159 reflections with *I* > 2σ(*I*)
graphite	*R*_int_ = 0.019
Rotation method data acquisition using ω and φ scans	θ_max_ = 26.4°, θ_min_ = 2.6°
Absorption correction: multi-scan (*CrysAlis RED*; Oxford Diffraction, 2009)	*h* = −8→7
*T*_min_ = 0.052, *T*_max_ = 0.197	*k* = −10→14
2304 measured reflections	*l* = −10→9

### Refinement

**Table:**

Refinement on *F*^2^	Primary atom site location: structure-invariant direct methods
Least-squares matrix: full	Secondary atom site location: difference Fourier map
*R*[*F*^2^ > 2σ(*F*^2^)] = 0.024	Hydrogen site location: inferred from neighbouring sites
*wR*(*F*^2^) = 0.062	H atoms treated by a mixture of independent and constrained refinement
*S* = 1.11	*w* = 1/[σ^2^(*F*_o_^2^) + (0.0387*P*)^2^ + 1.5899*P*] where *P* = (*F*_o_^2^ + 2*F*_c_^2^)/3
1240 reflections	(Δ/σ)_max_ = 0.049
55 parameters	Δρ_max_ = 2.00 e Å^−^^3^
3 restraints	Δρ_min_ = −1.60 e Å^−^^3^

### Special details

**Table:**

Experimental. CrysAlis RED (Oxford Diffraction, 2009) Empirical absorption correction using spherical harmonics, implemented in SCALE3 ABSPACK scaling algorithm.
Geometry. All e.s.d.'s (except the e.s.d. in the dihedral angle between two l.s. planes) are estimated using the full covariance matrix. The cell e.s.d.'s are taken into account individually in the estimation of e.s.d.'s in distances, angles and torsion angles; correlations between e.s.d.'s in cell parameters are only used when they are defined by crystal symmetry. An approximate (isotropic) treatment of cell e.s.d.'s is used for estimating e.s.d.'s involving l.s. planes.
Refinement. Refinement of *F*^2^ against ALL reflections. The weighted *R*-factor *wR* and goodness of fit *S* are based on *F*^2^, conventional *R*-factors *R* are based on *F*, with *F* set to zero for negative *F*^2^. The threshold expression of *F*^2^ > σ(*F*^2^) is used only for calculating *R*-factors(gt) *etc*. and is not relevant to the choice of reflections for refinement. *R*-factors based on *F*^2^ are statistically about twice as large as those based on *F*, and *R*- factors based on ALL data will be even larger.

### Fractional atomic coordinates and isotropic or equivalent isotropic displacement parameters (Å^2^)

**Table:**

	*x*	*y*	*z*	*U*_iso_*/*U*_eq_	
N1	0.8265 (7)	0.0383 (4)	0.2834 (5)	0.0106 (9)	
H1A	0.8539	−0.0264	0.2258	0.013*	
H1B	0.9046	0.0996	0.2595	0.013*	
H1C	0.6866	0.0563	0.2501	0.013*	
C1	0.8833 (8)	0.0144 (5)	0.4718 (6)	0.0118 (11)	
H11	0.7985	−0.0521	0.4984	0.014*	
H12	0.8511	0.0840	0.5349	0.014*	
Hg1	0.55745 (4)	0.2500	0.75798 (4)	0.01179 (11)	
Br1	0.69952 (8)	0.04665 (4)	0.85916 (6)	0.01038 (14)	
Br2	0.50796 (11)	0.2500	0.41588 (9)	0.01061 (17)	
Br3	0.15662 (11)	0.2500	0.79637 (10)	0.01234 (17)	
O1	0.0198 (8)	0.2500	0.1966 (7)	0.0125 (11)	
H1O	0.032 (9)	0.1920 (13)	0.127 (5)	0.015*	

### Atomic displacement parameters (Å^2^)

**Table:**

	*U*^11^	*U*^22^	*U*^33^	*U*^12^	*U*^13^	*U*^23^
N1	0.017 (2)	0.006 (2)	0.008 (2)	0.0032 (17)	0.0007 (18)	0.0019 (17)
C1	0.018 (3)	0.011 (2)	0.007 (2)	0.005 (2)	0.005 (2)	0.001 (2)
Hg1	0.01411 (17)	0.00870 (16)	0.01290 (17)	0.000	0.00384 (11)	0.000
Br1	0.0130 (3)	0.0098 (3)	0.0085 (2)	0.00092 (19)	0.00280 (19)	0.00217 (19)
Br2	0.0128 (3)	0.0093 (3)	0.0090 (3)	0.000	0.0010 (3)	0.000
Br3	0.0134 (4)	0.0072 (3)	0.0185 (4)	0.000	0.0079 (3)	0.000
O1	0.015 (3)	0.010 (3)	0.014 (3)	0.000	0.005 (2)	0.000

### Geometric parameters (Å, °)

**Table:**

N1—C1	1.495 (6)	C1—H12	0.9900
N1—H1A	0.9100	Hg1—Br1^ii^	2.5597 (6)
N1—H1B	0.9100	Hg1—Br1	2.5597 (6)
N1—H1C	0.9100	Hg1—Br2	2.6862 (8)
C1—C1^i^	1.515 (10)	Hg1—Br3	2.6923 (8)
C1—H11	0.9900	O1—H1O	0.881 (19)
			
C1—N1—H1A	109.5	N1—C1—H12	109.7
C1—N1—H1B	109.5	C1^i^—C1—H12	109.7
H1A—N1—H1B	109.5	H11—C1—H12	108.2
C1—N1—H1C	109.5	Br1^ii^—Hg1—Br1	130.16 (3)
H1A—N1—H1C	109.5	Br1^ii^—Hg1—Br2	105.823 (15)
H1B—N1—H1C	109.5	Br1—Hg1—Br2	105.823 (15)
N1—C1—C1^i^	109.7 (5)	Br1^ii^—Hg1—Br3	104.551 (15)
N1—C1—H11	109.7	Br1—Hg1—Br3	104.551 (15)
C1^i^—C1—H11	109.7	Br2—Hg1—Br3	103.08 (3)

Symmetry codes: (i) −*x*+2, −*y*, −*z*+1; (ii) *x*, −*y*+1/2, *z*.

### Hydrogen-bond geometry (Å, °)

**Table:**

*D*—H···*A*	*D*—H	H···*A*	*D*···*A*	*D*—H···*A*
N1—H1A···Br3^iii^	0.91	2.56	3.359 (5)	147
N1—H1A···Br1^i^	0.91	3.14	3.655 (5)	118
N1—H1B···O1^iv^	0.91	1.98	2.882 (5)	169
N1—H1C···Br1^iii^	0.91	2.72	3.482 (4)	141
N1—H1C···Br2	0.91	2.95	3.503 (5)	121
O1—H1O···Br3^v^	0.88 (2)	3.02 (3)	3.521 (6)	118 (2)

Symmetry codes: (iii) −*x*+1, −*y*, −*z*+1; (i) −*x*+2, −*y*, −*z*+1; (iv) *x*+1, *y*, *z*; (v) *x*, *y*, *z*−1.
